# Identification and molecular characterization of an IDA-like gene from litchi, *LcIDL1*, whose ectopic expression promotes floral organ abscission in *Arabidopsis*

**DOI:** 10.1038/srep37135

**Published:** 2016-11-15

**Authors:** Peiyuan Ying, Caiqin Li, Xuncheng Liu, Rui Xia, Minglei Zhao, Jianguo Li

**Affiliations:** 1 State Key Laboratory for Conservation and Utilization of Subtropical Agro-Bioresources, China Litchi Research Center, South China Agricultural University, Guangzhou, 510642, China; 2 Guangdong Litchi Engineering Research Center, College of Horticulture, South China Agricultural University, Guangzhou, 510642, China; 3 Key Laboratory of Plant Resources Conservation and Sustainable Utilization, South China Botanical Garden, Chinese Academy of Sciences, Guangzhou 510650, China

## Abstract

Unexpected abscission of flowers or fruits is a major limiting factor for crop productivity. Key genes controlling abscission in plants, especially in popular fruit trees, are largely unknown. Here we identified a litchi (*Litchi chinensis* Sonn.) IDA-like (INFLORESCENCE DEFICIENT IN ABSCISSION-like) gene *LcIDL1* as a potential key regulator of abscission. *LcIDL1* encodes a peptide that shows the closest homology to *Arabidopsis* IDA, and is localized in cell membrane and cytoplasm. Real-time PCR analysis showed that the expression level of *LcIDL1* accumulated gradually following flower abscission, and it was obviously induced by fruit abscission-promoting treatments. Transgenic plants expressing *LcIDL1* in *Arabidopsis* revealed a role of *LcIDL1* similar to *IDA* in promoting floral organ abscission. Moreover, ectopic expression of *LcIDL1* in *Arabidopsis* activated the expression of abscission-related genes. Taken together, our findings provide evidence that *LcIDL1* may act as a key regulator in control of abscission.

Abscission is characterized by a coordination of biochemical events that take place in abscission zones (AZ) to shed vegetative or reproductive organs^1^. Currently it is well known that abscission involves multiple changes in cell structure, metabolism and gene expression, and the process is divided into four major steps^1^,^2^: (i) the differentiation of AZ at a specific position, (ii) the acquisition by the AZ of competence, (iii) the onset of the cell separation within the AZ, (iv) the differentiation of a protective layer at the plant’s side. Over the past decades, a few genes have been found to play important roles in these four steps, which are mainly reported in model plants tomato and *Arabidopsis*^3^,^4^,^5^,^6^,^7^,^8^,^9^,^10^,^11^,^12^,^13^,^14^,^15^,^16^. In tomato, the MADS box transcriptional factors JOINTLESS and MACROCALYX form a complex to regulate the formation of the pedicel AZ together with the VHIID protein LATERAL SUPPRESSOR^11^,^17^. In *Arabidopsis*, two genes, *BLADEON-PETIOLE 1* and *2 (BOP1*, *BOP2*) which encode proteins belonging to a family containing BTB/POZ domains and ankyrin repeats, are necessary for AZ development. *bop1bop2* double mutants retain all floral organs indefinitely and do not form cytologically distinct and active floral AZ cells^7^. NtBOP2, a homologue of the *Arabidopsis* BOP2 protein, also showed a specific role in the differentiation of the corolla AZ^16^. When AZ differentiation is completed, phytohormones are thought to be important abscission signals. In general, ethylene and jasmonic acid accelerate abscission, while auxin, gibberellins, and brassinosteroids inhibit abscission^1^. In addition, many studies have shown that the rate and degree of abscission are largely dependent on the balance between the levels of auxin and ethylene in AZ, especially on changes in auxin gradients^10^,^18^,^19^,^20^. Once the abscission process is activated, many components start to function in the AZ. For the third phase, an IDA-HAESA (HAE)/HAESA-LIKE2 (HSL2) signaling system has been characterized during floral organ abscission in *Arabidopsis*^4^,^8^,^9^,^15^,^21^. Briefly, *IDA* encodes a peptide ligand that forms a complex with redundant receptor-like kinases HAE and HSL2, which presumably activates a MITOGEN-ACTIVATED PROTEIN KINASE (MAPK) cascade that acts to regulate downstream KNOX-like transcription factor BP/KNAT1. Recently, it has been proposed that the involvement of this signaling module in abscission processes is conserved in other plant species^22^,^23^. However, so far, the characterization of key regulators in control of abscission has been reported mainly in the model plants tomato and *Arabidopsis*; these factors are largely unknown in perennial woody plants, especially in important horticultural cultivated fruit crops, such as litchi.

Litchi (*Litchi chinensis* Sonn.), a famous fruit tree originating from South China, has been widely cultivated in Southeast Asia due to its delicious and nutritional fruits. The litchi tree is easily subject to massive fruit drop, leading to low yield and heavy economic loss^24^,^25^. For example, a medium-sized tree may produce close to 60,000 female flowers but less than 5% of flowers can develop into mature fruits^26^,^27^. There are three to four waves of physiological fruit drop throughout fruit development depending on cultivar. Wave I, wave II, and wave III of abscission occur around 1 week, 3 weeks, and 6–7 weeks after full bloom, respectively, but wave IV is cultivar-specific and occurs at 2–3 weeks before harvest^26^,^27^. Thus, to reduce unexpected fruit abscission in litchi, a number of studies have been conducted on endogenous hormones^27^,^28^,^29^ and carbohydrates^30^,^31^,^32^,^33^,^34^,^35^, which are proposed to play vital roles in the regulation of fruit abscission. Recently, genome-wide digital transcript analysis further revealed that a range of fruitlet abscission-related genes are regulated by ethylene and carbohydrate stress^36^,^37^. In fact, massive transcriptomic and metabonomic data about abscission were also obtained in other fruit trees, such as citrus leaf and shoot tips^38^,^39^, apple young fruits^40^,^41^ and olive mature fruits^42^. A deeper understanding of the mechanisms underlying abscission would be helpful not only for the regulation of fruit crop production and the improvement of current cultural and management practices, but also for the elucidation of new molecular markers to improve genetic breeding^43^.

Here we identified three IDA-like genes in litchi, of which *LcIDL1* shows the closest homology to *Arabidopsis* IDA and is found to be involved in litchi male flower and fruitlet abscission. Ectopic expression of *LcIDL1* in *Arabidopsis* revealed that *LcIDL1* is able to function to induce floral organ abscission. Our data suggest that *LcIDL1* may act as a key regulator in control of abscission.

## Results

### Identification of key candidate genes regulating fruit abscission in litchi

Previously, in an effort to identify the components of fruit abscission in litchi, we screened for genes that were differentially expressed during abscission from a RNA-seq database. Through GO (Gene Ontology) and KEGG (Kyoto Encyclopedia of Genes and Genomes) pathway analysis, a number of genes were identified as the candidate components involved in fruit abscission process. These genes were clustered into diverse metabolic processes and pathways, including carbohydrate metabolism, plant hormone synthesis and signaling, transcription factor activity and cell wall modification^36^,^37^. But which is a master one? We found *Litchi_GLEAN_10054315* encoding an *IDA (INFLORESCENCE DEFICIENT IN ABSCISSION*)-like gene and being highly expressed before fruit abscission. Here we designated it as *LcIDL1*. It appears that key regulators, such as IDA/IDL, may have conserved function in control of cell separation in different plant species^4^, so *LcIDL1* attracted much more attention in our lab.

### *LcIDL1* is an *IDA*-like gene and shows closest homology to *Arabidopsis IDA*

**Figure 1:**
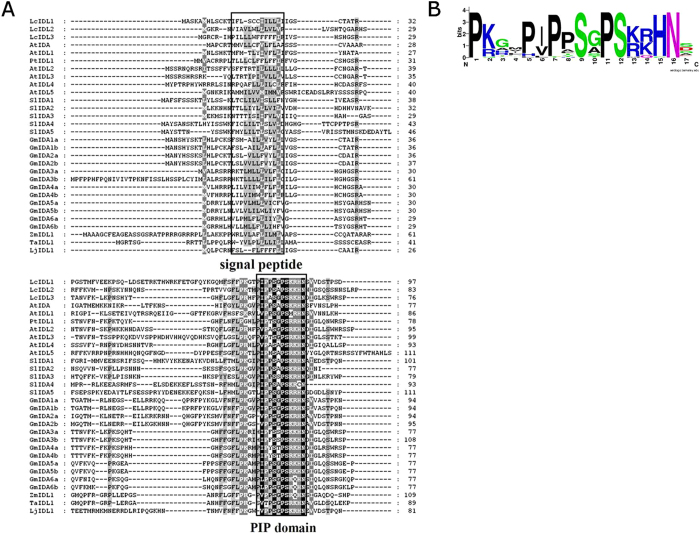
Alignment of LcIDLs with other plant IDA/IDL proteins. (**A**) Alignment was made using the ClustalW2 program (http://www.ebi.ac.uk/Tools/clustalw2/) with manual adjustment using GeneDoc (http://www.psc.edu/biomed/genedoc/). The amino acid sequences of LcIDL1 (Litchi_GLEAN_10054315), LcIDL2 (Litchi_GLEAN_10027620), LcIDL3 (Litchi_GLEAN_10009234), AtIDA (At1g68765), AtIDL1 (AT3G25655), AtIDL2 (AT5G64667), AtIDL3 (AT5G09805), AtIDL4 (AT3G18715), AtIDL5 (AT1G76952), PtIDL1 (BU889756), ljIDL1(AW719486), ZmIDL1(BI430572), TaIDL1 (BM135459), SlIDAs and GmIDAs (see reference Tucker and Yang, 2012) were aligned. The conserved domains of signal peptide and PIP are marked with rectangles. (**B**) A sequence logo representation of the conserved C-terminal PIP domain of IDA/IDL peptides.

To acquire all the *IDA*-like gene members in litchi, we used the conserved domain of IDA to blast against the litchi genome database. We obtained two more *IDA*-like genes, *Litchi_GLEAN_10027620* and *Litchi_GLEAN_10009234*, and we named them as *LcIDL2* and *LcIDL3*. IDA protein alignment between litchi and other plant species was conducted based on their full-length protein sequences. As shown in Fig. 1A, all members of the *IDL* gene family are characterized by an N-terminal secretory signal peptide (SP), a variable region and a conserved C-terminal region called the PIP domain^4^. The N-terminal of SP contains a stretch of aliphatic residues, a typical motif of secreted proteins. A sequence consensus logo of the C-terminal conserved PIP domain showed that this domain consists of a PSGPS motif and four highly conserved residues [(R/K)(R/K)HN] (Fig. 1B).

**Figure 2:**
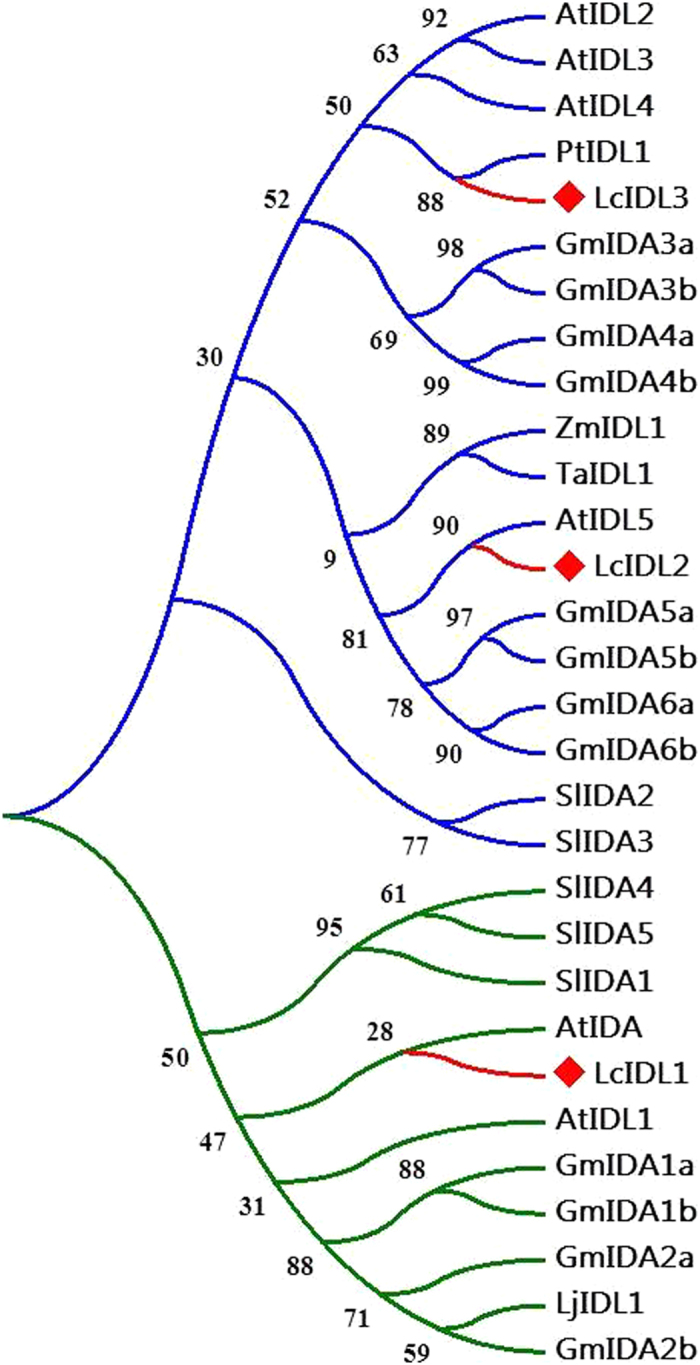
Phylogenetic tree of IDA/IDL proteins. A maximum likelihood phylogenetic tree was constructed based on the protein alignment of the IDA/IDL from *Arabidopsis (At*), tomato (*Sl*), lotus (*Lj*), soybean (*Gm*), maize (*Zm*), poplar (*Pt*) and wheat (*Ta*). The bootstrap consensus tree was inferred from 1000 replicates. The two top-level sub-branches are represented by blue and green color, respectively. The red lines and diamonds indicate the LcIDLs.

In order to examine the relationship of litchi *IDL* genes with those in other species, a phylogenetic analysis using the full-length IDA/IDL protein sequences from *Arabidopsis (At*), tomato (*Sl*), lotus (*Lj*), soybean (*Gm*), maize (*Zm*), poplar (*Pt*) and wheat (*Ta*) was performed. As shown in Fig. 2, the IDL family is divided into two subgroups, one containing LcIDL1 and the other containing LcIDL2 and LcIDL3. In contrast to LcIDL2 and LcIDL3, LcIDL1 shows the closest relationship to *Arabidopsis* IDA.

**Figure 3:**
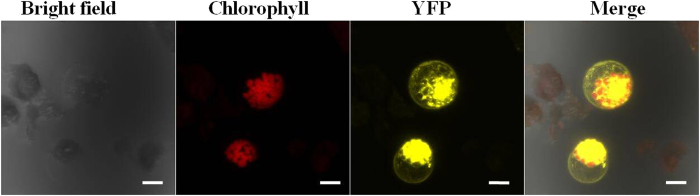
Subcellular localization analysis of LcIDL1. Constructs of LcIDL1-YFP were transfected into *Arabidopsis* protoplasts; and the fluorescence signal was detected with the laser scanning confocal microscope. The yellow color indicates fluorescence of YFP, while the red color indicates the auto-fluorescence of chlorophyll; the bar is 25 μm.

To investigate the subcellular localization of the LcIDL1 protein, the full-length coding sequence of *LcIDL1* fused with yellow fluorescent protein (YFP) was delivered into the *Arabidopsis* protoplasts. LcIDL1-YFP protein was observed to be localized to the cell membrane and cytoplasm (Fig. 3).

### *LcIDL1* is an abscission-associated gene during the male flower and fruitlet drop in litchi

**Figure 4:**
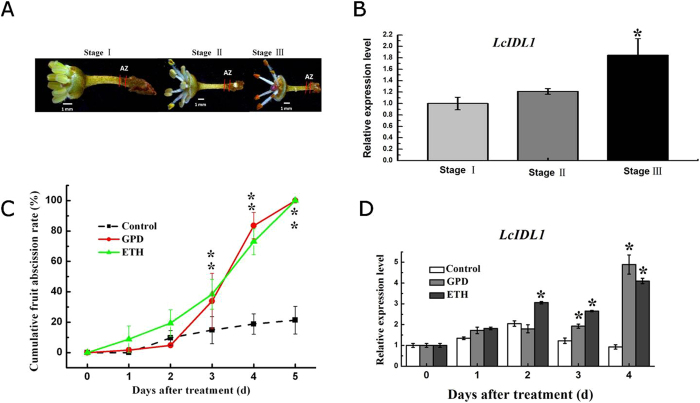
*LcIDL1* is an abscission-associated gene in litchi. (**A**) Magnified images of stage I, stage II and stage III of litchi male flower; the region between two short red lines indicates the AZ. (**B**) qRT-PCR analysis of *LcIDL1* expression in pedicel AZ cells of litchi male flower during abscission. The Y-axis is fold-change, the expression levels are relative to stage I. (**C**) Girdling plus defoliation (GPD) and ethephon (ETH) treatments significantly induced fruitlet abscission. (**D**) qRT-PCR analysis of *LcIDL1* expression in peduncle AZ cells after GPD and ETH treatments. *LcEF-1a* was used as an internal control. The Y-axis is fold-change, the expression levels are relative to control 0 d. Data shown are means ± SD. One-way ANOVA (Tukey-Kramer test) analysis was performed, and statistically significant differences (P < 0.05) were indicated by asterisks.

The expression level of *LcIDL1* in pedicel AZ tissues of male flowers was determined using a real-time PCR assay. Generally, the male flowers will drop at the pedicel AZ within one week after the stamens protruding out. AZ tissues of 1st day (stage I), 3rd day (stage II) and 7th day (stage III) after stamens protruding out were harvested for analysis (Fig. 4A). The transcription level of *LcIDL1* at stage III was increased significantly compared to the other two stages (Fig. 4B), which was consistent with the degree of flower abscission. Next, we examined the effect of two abscission-inducing treatments, girdling plus defoliation (GPD) and ethephon (ETH)^37^ on the transcription of *LcIDL1* during litchi fruitlet abscission. Both GPD and ETH treatments significantly promoted the fruitlet drop starting from the third day, the cumulative fruit abscission rate was up to 100% on the fifth day (Fig. 4C). The expression level of *LcIDL1* in pedicel AZ tissues was significantly induced after GPD and ETH treatments, with about 5-fold and 4-fold higher in GPD and ETH-treated AZ tissues than that in control on the fourth day (Fig. 4D). Collectively, these data suggested that *LcIDL1* is an abscission-associated gene in litchi.

### Ectopic expression of *LcIDL1* driven by the *35S* promoter strongly activated the floral organ abscission in *Arabidopsis*

**Figure 5:**
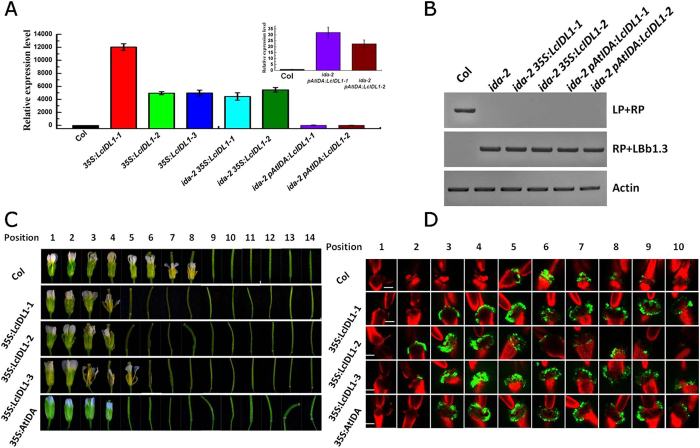
Overexpression of *LcIDL1* caused earlier floral organ abscission in *Arabidopsis*. (**A**) Expression level of *LcIDL1* in different transgenic *Arabidopsis* lines. *35S:LcIDL1-1*, *35S:LcIDL1-2*, and *35S:LcIDL1-3* lines were ectopic expression of *LcIDL1* under the control of the 35S promoter in wild type Col; *ida-2 35S:LcIDL1-1* and *ida-2 35S:LcIDL1-2* lines were ectopic expression of *LcIDL1* under the control of the 35S promoter in *ida-2*; *ida-2 pAtIDA:LcIDL1-1* and *ida-2 pAtIDA:LcIDL1-2* lines were ectopic expression of *LcIDL1* under the control of the *Arabidopsis IDA* promoter in *ida-2. AtUBQ* was used as an internal control for qRT-PCR analysis. The Y-axis is fold-change, the expression levels are relative to wild type Col. Data shown are means ± SD. (**B**) Genotyping analysis of IDA in Col, *ida-2*, *ida-2 35S:LcIDL1-1*, *ida-2 35S:LcIDL1-1*, *ida-2 pAtIDA:LcIDL1-1* and *ida-2 pAtIDA:LcIDL1-1*. The T-DNA insertion mutant *ida-2* are analyzed with LP (Left border primer of the T-DNA insertion), RP (Right border primer of the T-DNA insertion), and LBb1.3 (used for Salk genotyping project). (**C**) Phenotype of floral organ abscission in transgenic lines. Position numbers were counted from the first flower with visible white petals on the top of the inflorescence. (**D**) BCECF fluorescence micrographs of floral organ AZ of *Arabidopsis* Col*, 35S:LcIDL1-1*, *35S:LcIDL1-2*, and *35S:LcIDL1-3.* Inflorescences were sampled separately, incubated in BCECF solution, and examined by a confocal laser scanning microscope. The microscopic fluorescence images represent merged images of BCECF fluorescence with chlorophyll autofluorescence images. The increase in pH is shown by green fluorescence, which is distinguished from the red chlorophyll autofluorescence. Scale bars are 100 μm in length. The images presented for each plant and positions are representative images out of 3–4 replicates.

To examine the possible role of LcIDL1 in abscission, LcIDL1 was ectopically expressed in the model plant *Arabidopsis*. We constructed transgenic plants expressing *LcIDL1* under the control of the 35S promoter by *Agobacterium*-mediated plant transformation^44^. Meanwhile, transgenic *Arabidopsis* plants over-expressing *AtIDA* were also generated and used as a control. Homozygous *35S:LcIDL1* and *35S:IDA* transgenic lines were obtained by antiabiotic selection. Further, real-time PCR analysis showed that the transcript of *LcIDL1* was highly expressed in these transgenic plants (Fig. 5A). The phenotypes of the flowers or siliques at specific positions along the inflorescence of wild-type Col, *35S:LcIDL1*, and *35S:IDA* transgenic plants were examined. Similar to what has been reported previously^6^, the *35S:AtIDA* plants exhibited earlier abscission of floral organs (Fig. 5C), suggesting our construct is valid. Interestingly, we showed that the *35S:LcIDL1* plants abscised their flowers first at position 5, whereas the wild-type dropped their flowers first at position 9 (Fig. 5C), which suggested that *LcIDL1* is able to induce floral organ abscission in transgenic *Arabidopsis*.

Previous work revealed that the cytosolic pH increase in AZ cells occurs concomitantly with the execution of organ abscission^45^. We further observed the pH-value in the abscission zone of the floral organs using a pH-sensitive indicator, BCECF^45^. The BCECF-treated floral organs were imaged using a confocal microscope. As shown in Fig. 5D, obvious green fluorescence was observed in the abscission zone of the position 5 flowers in the wild-type. In contrast, the *35S:LcIDL1* and *35S:AtIDA* overexpression lines not only displayed much earlier BCECF green fluorescence which can be detected at P3, but also showed much stronger BCECF green fluorescence and maintained a relatively higher signal density prolonged to position 10 (Fig. 5D). These data confirmed that *LcIDL1* is able to function to promote floral organ abscission in *Arabidopsis*.

### *LcIDL1* has a similar role to *AtIDA* in control of floral organ abscission in *Arabidopsis*

**Figure 6:**
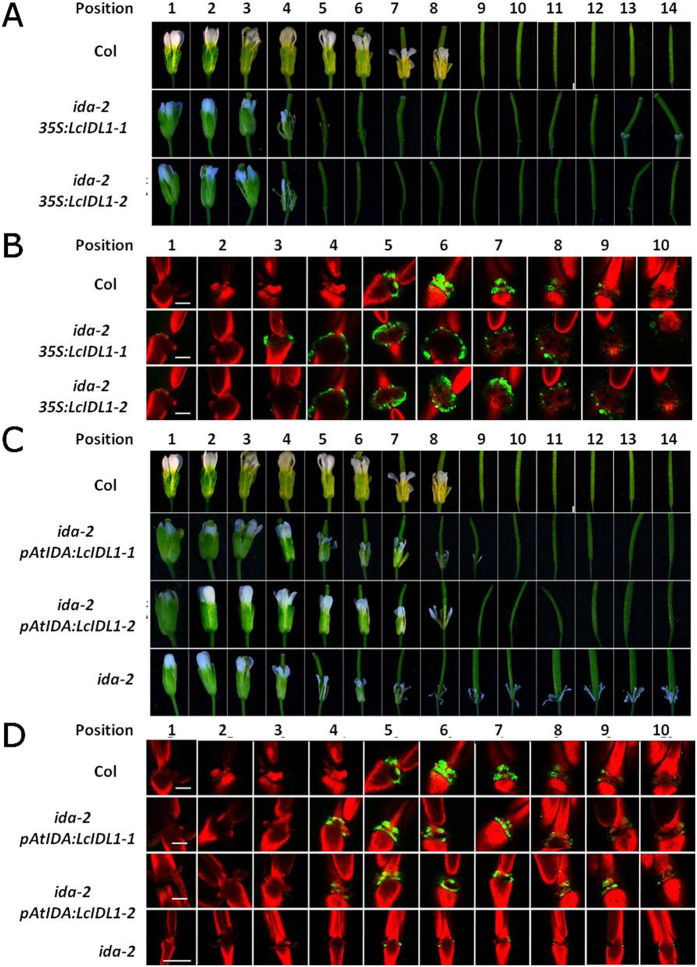
*LcIDL1* has a similar role as *AtIDA* in control of floral organ abscission. (**A**) *ida-2 35S:LcIDL1-1 and ida-2 35S:LcIDL1-2* displayed earlier floral organ abscission. Position numbers were counted from the first flower with visible white petals on the top of the inflorescence. (**B**) BCECF fluorescence micrographs of floral organ AZ of *Arabidopsis* Col*, ida-2 35S:LcIDL1-1* and *ida-2 35S:LcIDL1-2.* Scale bars are 100 μm. The images presented for each plant and positions are representative images out of 3–4 replicates. (**C**) *ida-2 pAtIDA:LcIDL1-1* and *ida-2 pAtIDA:LcIDL1-2* displayed normal floral organ abscission. Position numbers were counted from the first flower with visible white petals on the top of the inflorescence. (**D**) BCECF fluorescence micrographs of floral organ AZ of *Arabidopsis* Col*, ida-2 pAtIDA:LcIDL1-1* and *ida-2 pAtIDA:LcIDL1-2.* Scale bars are 100 μm. The images presented for each plant and position are representative images out of 3–4 replicates.

Phylogenetic analysis revealed that LcIDL1 is a close homologue of AtIDA. To further compare the role of *LcIDL1* and *AtIDA* in floral organ abscission, we generated transgenic plants expressing *LcIDL1* driven by 35S or *AtIDA* promoters under an *ida-2* mutant background^9^. Homozygous *ida-2 35S:LcIDL1* and *ida-2 pAtIDA:LcIDL1* transgenic lines were selected and validated by real-time PCR analysis (Fig. 5A,B). As described previously^9^, *ida-2*, the T-DNA insertion allele of *ida*, is deficient in floral organ abscission (Fig. 6C). We showed that the floral organs of *ida-2 35S:LcIDL1–1* and *ida-2 35S:LcIDL1-2* dropped at position 5, which is earlier than the wild-type Col (position 9) and *ida-2* mutant (Fig. 6A). Consistently, the BCECF green fluorescence in AZ cells of *ida-2 35S:LcIDL1* plants was observed earlier, stronger and longer than that in Col plants (Fig. 6B).

Furthermore, we showed that *ida-2 pAtIDA:LcIDL1* transgenic lines *ida-2 pAtIDA:LcIDL1-1* and *ida-2 pAtIDA:LcIDL1-2* displayed the same floral organ abscission process as the wild-type, with the floral organs dropped at position 9 (Fig. 6C). In addition, the BCECF green fluorescence in AZ cells of *ida-2 pAtIDA:LcIDL1-1* and *ida-2 pAtIDA:LcIDL1-2* transgenic lines was also recovered (Fig. 6D), which suggested that ectopic expression of *LcIDL1* driven by the *Arabidopsis IDA* promoter completely rescued the *ida-2* floral organ abscission phenotype. Collectively, these data confirmed that *LcIDL1* is capable of functioning in place of *AtIDA* to induce floral organ abscission in *Arabidopsis*.

### Ectopic expression of *LcIDL1* in *Arabidopsis* activated abscission-related gene expression

**Figure 7:**
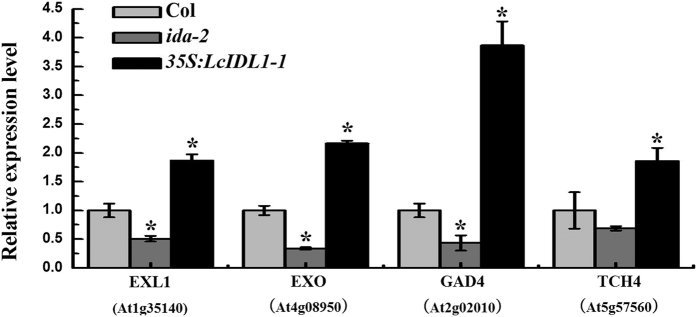
Expression levels of abscission-related cell wall remodeling genes in Col, *ida-2* and *35S:LcIDL1-1*. AZ tissues of position 3–8 were collected for testing. *AtUBQ* was used as an internal control for qRT-PCR analysis. The Y-axis is fold-change, the expression levels are relative to wild type Col. Data shown are means ± SD. One-way ANOVA (Tukey-Kramer test) analysis was performed, and statistically significant differences (P < 0.05) were indicated by asterisks.

A previous study reported a positive role of IDA in regulating abscission-related gene expression^14^. In the present work, the expression levels of a subset of abscission-related genes, such as cell-wall remodelling (*TCH4*/*XTH22*)^3^, cell expansion (*EXO* and *EXL1*)^46^, Ca^2+^ binding (GAD4)^47^ were tested with qRT-PCR in Col, *35S:LcIDL1-1* and *ida-2*. AZ regions undergoing cell-wall loosening and organ separation (positions 3–8) were harvested for analysis. As shown in Fig. 7, the expression level of *TCH4*, *EXO*, *EXL1* and *GAD4* was decreased in *ida-2* compared with wild-type. In contrast, the transcription of these genes was significantly up-regulated in *35S:LcIDL1-1* compared with the wild-type. These data suggested that LcLDL1 may induce floral organ abscission by activating abscission-related gene expression.

## Discussion

To date, *IDA-*like genes have been found in *Arabidopsis*^15^, soybean (*Glycine max*), tomato (*Solanum lycopersicum*)^12^ and citrus (sweet orange and clementine)^22^. However, only *Arabidopsis* IDA has been tested for subcellular localization and found localized in the cell membrane, supporting that IDA, as a signal peptide, can be secreted^4^. In our report, LcIDL1 can be detected in the cytoplasm (Fig. 3). AtIDA is, in contrast to LcIDL1, not found in the cytoplasm. One possible reason is that AtIDA and LcIDL1 do not function completely similarly, the other one is that different systems were used for signal detection, since AtIDA was bombarded into the onion cells and *Arabidopsis* protoplasts were transfected in the present assay. Thus further study on IDA delivered to *Arabidopsis* protoplasts can tell more.

That ethylene acts an important factor inducing organ shedding has been established for many years^48^. Exposure to exogenous ethylene has no impact on the inhibition of floral organ abscission and ethylene sensitivity in *ida* mature plants, suggesting that IDA acts downstream of ethylene in the pathway by which ethylene controls abscission^4^. Consistent with this, the promoter fusion construct *IDA:GUS*, which is primarily expressed in the floral AZ and floral organs, is restricted only to the nectaries in ethylene insensitive mutant *etr1-1*, indicating that ethylene acts at least partly through ETR1 to transcriptionally induce IDA expression which then instigates abscission. Here our report further supports that *IDA/IDL* genes seem to be influenced by an ethylene-response pathway. When treated with ethylene, the expression level of *LcIDL1* significantly increased in AZ cells. Additionally, GPD treatment induced much higher expression of *LcIDL1* (Fig. 4), we inferred that this is likely also associated with ethylene emission induced by GPD treatment^36^. Similar results were also obtained in *Populus* and oil palm, which showed that *PtIDA* and *PtIDL1* were expressed more highly in leaves after shade treatment (a similar treatment to GPD resulting in carbohydrate stress), and *EgIDA2* and *EgIDA5* could be induced by ethylene^23^, further supporting that IDA functions in the ethylene-response pathway.

Genetic studies have already revealed a framework of cell signalling, membrane trafficking, and transcriptional networks in the later stages of floral organ abscission in *Arabidopsis*, of which IDA has been found as a critical regulator as *ida* mutant plants are deficient in floral organ abscission in *Arabidopsis*^4^, while opposite to what is observed in the *ida* mutant, *Arabidopsis* plants overexpressing *IDA* cause precocious floral organ abscission^6^. Interestingly, transgenic plants expressing *LcIDL1* in *Arabidopsis* also caused earlier floral organ abscission, suggesting that *LcIDL1* is able to function to induce floral abscission in *Arabidopsis* (Fig. 5). In addition, beyond our expectation, transgenic plants expressing *LcIDL1* under the control of the 35S promoter in *ida-2* also displayed earlier floral organ abscission (Fig. 6), suggesting a similar role of LcIDL1 to AtIDA. More importantly, transgenic plants expressing *LcIDL1* under the control of the *Arabidopsis* IDA native promoter in *ida-2* can completely restore the deficiency in floral organ abscission, further supporting the notion that LcIDL1 shares the same function with AtIDA of promoting floral organ abscission in *Arabidospsis* (Fig. 6). Similarly, that citrus *CitIDA3* is able to function to promote floral organ abscission in transgenic *Arabidopsis* has also been reported, and ectopic expression of *CitIDA3* could also complement the abscission deficiency of the *ida* mutant^22^. Taken together, these results indicate that IDA, as a regulator of abscission, is functionally conserved in different species.

It has been suggested that pH changes in AZ cells are associated with abscission progress. Before onset of organ abscission, the alkalization of the cytosol in AZ cells will occur and stronger BCECF fluorescence will be detected in AZ cells of mutants that promote abscission. Mutants that inhibit abscission will maintain a low pH value and weak BCECF fluorescence in AZ cells^45^. In the present study, we showed that an earlier, stronger and longer BCECF fluorescence can be detected in transgenic plants overexpressing *LcIDL1* which displayed significantly earlier floral organ abscission (Figs 5 and 6), indicating that there is a strong correlation between pH changes in AZ cells and the abscission process. Further, genes involved in pH changes in AZ cells need to be identified.

Once the abscission process is activated, the pectin in the cell walls between the AZ cell layers starts to degrade followed by an expansion in the size of the AZ cells. A concerted effort of research on many plant species has identified cell wall remodeling enzymes acting on structural polysaccharides leading to the hydrolysis of the middle lamella and cell walls of the AZ cells^1^. It has also revealed that IDA not only regulates floral abscission but also regulates lateral root emergence through changing the CWR enzyme activity^13^. In this study, higher expression levels of four abscission-related cell wall remodeling genes, *GAD4*, *TCH4*, *EXO* and *EXL1*, were detected in *LcIDL1* overexpression lines (Fig. 7). In addition, our previous report showed that a cluster of cell wall degradation and loosening genes including **β**-1,3-glucanase, **β**-D-xylosidase, endoglucanase, xyloglucan endotransglucosylase/hydrolase, pectinesterase, polygalacturonase, and **β**-D-xylosidase were induced in AZ cells during litchi fruit abscission. Collectively, it can be proposed that IDA/IDL regulates initial cell wall loosening and separation of AZ cells to induce abscission, probably by controlling the expression of cell wall remodelling genes.

In conclusion, our findings revealed that LcIDL1 is likely involved in the regulation of male flower and fruitlet abscission in litchi. Furthermore, LcIDL1 functions similarly as AtIDA to promote floral organ abscission in *Arabidopsis*, which may operate through regulating pH changes and cell wall remodelling genes in AZ cells.

## Materials and Methods

### Plant materials and treatments

For litchi, three 9-year-old litchi trees (*Litchi chinensis* Sonn. cv. Feizixiao) grown in an orchard in South China Agricultural University (Guangzhou, China) were randomly selected. Thirty fruit bearing shoots with similar diameter (about 5–8 mm) growing in different directions from each tree were tagged. Ten of them were treated with girdling (a ring of bark about 0.5 cm in width and cambium was removed from the branch base) followed by defoliation (removing all leaves above the girdle) at 35 days after anthesis (GPD treatment); Ten of them were dipped in 250 mg L^−1^ ethephon solution (containing 0.05% Tween-80 surfactant) for 1 min (ETH treatment), while the remaining untreated shoots were used as controls. Three out of each set of ten treated shoots were used to monitor fruit abscission dynamics for 5 days and the others were used for sampling. Cumulative fruit abscission rate (CFAR) was calculated according to our previous method^49^. Samples were collected at 0, 1, 2, 3 and 4 days after treatment. AZ was excised by cutting around 2 mm at each side of the abscission fracture plane. After separation, all tissues were quickly frozen in liquid nitrogen and stored at −80 °C for future analysis. Each tree was treated as a biological replicate. For the flower assay, male flowers were selected and flowers with stamens just spreading were designed as stage I, the 3rd day after stamens spreading was designed as stage II, and the 7th day after stamens spreading was designed as stage III. Male flower AZ tissues were sampled as mentioned above.

For *Arabidopsis* materials, *Arabidopsis* ecotype Col-0 was used in all experiments and the *ida-2* mutant (SALK_133209)^9^, which is in the Col background, was obtained from the Nottingham *Arabidopsis* Stock Centre (NASC). For generation of *LcIDL1* and *AtIDA* overexpression lines in Col or in *ida-2*, the full-length open reading frame (ORF) of *LcIDL1* and AtIDA were subcloned into vector pCAMBIA1302 under the control of the *35S* promoter with Clontech’s In-Fusion system primers (Supplemental Table), then these constructs were transformed into Col or *ida-2* plants following the floral dip method^44^. For generation of *ida-2 pAtIDA:LcIDL1* transgenic plants, the full-length ORF of *LcIDL1* was subcloned into vector pCAMBIA1302 with the 35S promoter replaced with the *Arabidopsis IDA* promoter (−1 to −1490 bp), then this vector was transformed into *ida-2* plants following the floral dip method. The T3 homozygous transgenic plants were used for phenotypic analysis. All the *Arabidopsis* plants were grown at 22 °C under long-day (16 h light/8 h dark) conditions. To reduce variation, all genotypes tested in each experiment were grown together.

### Subcellular Localization Analysis

The coding sequence of LcIDL1 without the stop codon was amplified by PCR primers (listed in Supplemental Table) and then subcloned into the pSAT6-EYFP-N1 vector and fused in-frame with the Yellow Fluorescent Protein (YFP) sequence under the control of the *35S* promoter. The fusion constructs were introduced into *Arabidopsis* protoplasts by using 40% polyethylene glycol (PEG) as described previously^50^. YFP fluorescence was observed with a confocal laser scanning microscope (LSM 7 DUO, ZEISS, Germany). The transient expression assay was repeated three times.

### Quantitative RT-PCR Analysis

Total RNA was isolated from litchi AZ tissues or *Arabidopsis* leaves (20-day-old) using 1 mL Trizol reagent (Invitrogen). The first strand cDNA synthesis was generated using 2 μg total RNA according to the manufacturer’s instructions of the TransScript One-Step gDNA Removal and cDNA Synthesis SuperMix Kit (TransGen, Beijing). 100 ng synthesized cDNA was used as a template to perform quantitative RT-PCR analysis. PCR reactions were performed in a total volume of 20 μL, with 0.5 μL for each primer (10 mM, final concentration 100 nM) and 10 μL for SYBR Green PCR Supermix (Bio-Rad) on an ABI7500 Real-Time PCR System (Applied Biosystems). The PCR program included an initial denaturation step at 94 °C for 3 min, followed by 40 cycles of 5 s at 94 °C and 1 min at 60 °C. Each sample was quantified at least in triplicate and normalized using *Ubiquitin 10 (UBQ*) or *EF-1a* as an internal control for *Arabidopsis* and litchi^51^, respectively. The gene-specific primer pairs for quantitative Real-Time PCR are listed in Supplemental Table. All PCR reactions were normalized using a C_t_ value corresponding to the reference gene. The relative expression levels of target gene were calculated with the formula 2^−ddCt^ 52. Values represented the average of three biological replicates.

### Phenotypic Analysis

Litchi and *Arabidopsis* flowers were carefully removed from the plant body and then were imaged using a stereoscope (ZEISS, SV11). The whole *Arabidopsis* plant and a single leaf placed on a black cloth were photographed by digital camera (D3200, Nikon).

### BCECF fluorescence analyses

BCECF fluorescence analysis was conducted according to a previous detailed method with some modification^45^. Inflorescences with flowers located at various positions along the inflorescence were carefully removed from the plant body and immersed in 10 μM BCECF-AM (B1150, Thermo Scientific™) solution under darkness for 20 min, then the inflorescences were rinsed four times with phosphate-buffered saline (PBS, pH 7.4) to remove excess BCECE-AM. Before imaging, flowers at different positions were excised separately from the inflorescences, and each flower’s sepals, petals, and stamens were removed using forceps without damaging the carpels, receptacle, and peduncle. The images were snapped with a confocal laser scanning microscope (LSM 7 DUO, ZEISS, Germany). Samples were excited by both 488 nm and 633 nm light, then BCECF fluorescence and chlorophyll auto-fluorescence were detected through 494–598 and 647–721 filters, respectively. All BCECF experiments were repeated three times with different biological samples from different inflorescences, and representative images are presented.

## Additional Information

**How to cite this article**: Ying, P. *et al.* Identification and molecular characterization of an IDA-like gene from litchi, *LcIDL1*, whose ectopic expression promotes floral organ abscission in *Arabidopsis.* *Sci. Rep.* **6**, 37135; doi: 10.1038/srep37135 (2016).

**Publisher’s note:** Springer Nature remains neutral with regard to jurisdictional claims in published maps and institutional affiliations.

## Supplementary Material

Supplementary Information

## References

[b1] Estornell L. H., Agusti J., Merelo P., Talon M. & Tadeo F. R. Elucidating mechanisms underlying organ abscission. Plant Sci. 199, 48–60 (2013). 23265318 10.1016/j.plantsci.2012.10.008

[b2] Patterson S. E. Cutting loose. Abscission and dehiscence in Arabidopsis. Plant Physiol. 126, 494–500 (2001). 11402180 10.1104/pp.126.2.494 PMC1540116

[b3] Xu W. *et al.* Arabidopsis TCH4, regulated by hormones and the environment, encodes a xyloglucan endotransglycosylase. Plant Cell 7, 1555–1567 (1995). 7580251 10.1105/tpc.7.10.1555 PMC161010

[b4] Butenko M. A. *et al.* Inflorescence deficient in abscission controls floral organ abscission in Arabidopsis and identifies a novel family of putative ligands in plants. Plant Cell 15, 2296–2307 (2003). 12972671 10.1105/tpc.014365 PMC197296

[b5] Ellis C. M. *et al.* AUXIN RESPONSE FACTOR1 and AUXIN RESPONSE FACTOR2 regulate senescence and floral organ abscission in Arabidopsis thaliana. Development 132, 4563–4574 (2005). 16176952 10.1242/dev.02012

[b6] Stenvik G. E., Butenko M. A., Urbanowicz B. R., Rose J. K. & Aalen R. B. Overexpression of INFLORESCENCE DEFICIENT IN ABSCISSION activates cell separation in vestigial abscission zones in Arabidopsis. Plant Cell 18, 1467–1476 (2006). 16679455 10.1105/tpc.106.042036 PMC1475485

[b7] McKim S. M. *et al.* The BLADE-ON-PETIOLE genes are essential for abscission zone formation in Arabidopsis. Development 135, 1537–1546 (2008). 18339677 10.1242/dev.012807

[b8] Stenvik G. E. *et al.* The EPIP peptide of INFLORESCENCE DEFICIENT IN ABSCISSION is sufficient to induce abscission in arabidopsis through the receptor-like kinases HAESA and HAESA-LIKE2. Plant Cell 20, 1805–1817 (2008). 18660431 10.1105/tpc.108.059139 PMC2518227

[b9] Cho S. K. *et al.* Regulation of floral organ abscission in Arabidopsis thaliana. Proc. Natl. Acad. Sci. 105, 15629–15634 (2008). 18809915 10.1073/pnas.0805539105 PMC2563077

[b10] Meir S. *et al.* Microarray analysis of the abscission-related transcriptome in the tomato flower abscission zone in response to auxin depletion. Plant Physiol. 154, 1929–1956 (2010). 20947671 10.1104/pp.110.160697 PMC2996037

[b11] Nakano T. *et al.* MACROCALYX and JOINTLESS interact in the transcriptional regulation of tomato fruit abscission zone development. Plant Physiol. 158, 439–450 (2012). 22106095 10.1104/pp.111.183731 PMC3252084

[b12] Tucker M. L. & Yang R. IDA-like gene expression in soybean and tomato leaf abscission and requirement for a diffusible stelar abscission signal. Aob Plants 2012, pls035–pls035 (2012). 23585923 10.1093/aobpla/pls035 PMC3624929

[b13] Kumpf R. P. *et al.* Floral organ abscission peptide IDA and its HAE/HSL2 receptors control cell separation during lateral root emergence. Proc. Natl. Acad. Sci. 60, 2289–2296 (2013). 10.1073/pnas.1210835110 PMC3612645 23479623

[b14] Liu B. *et al.* NEVERSHED and INFLORESCENCE DEFICIENT IN ABSCISSION are differentially required for cell expansion and cell separation during floral organ abscission in Arabidopsis thaliana. J. Exp. Bot. 64, 5345–5357 (2013). 23963677 10.1093/jxb/ert232

[b15] Vie A. K. *et al.* The IDA/IDA-LIKE and PIP/PIP-LIKE gene families in Arabidopsis: phylogenetic relationship, expression patterns, and transcriptional effect of the PIPL3 peptide. J. Exp. Bot. 66, 5351–5365 (2015). 26062745 10.1093/jxb/erv285 PMC4526919

[b16] Wu X. M. *et al.* The tobacco BLADE-ON-PETIOLE2 gene mediates differentiation of the corolla abscission zone by controlling longitudinal cell expansion. Plant Physiol. 159, 835–850 (2012). 22492844 10.1104/pp.112.193482 PMC3375945

[b17] Schumacher K., Schmitt T., Rossberg M., Schmitz G. & Theres K. The Lateral suppressor (Ls) gene of tomato encodes a new member of the VHIID protein family. Proc. Natl. Acad. Sci. 96, 290–295 (1999). 9874811 10.1073/pnas.96.1.290 PMC15132

[b18] Patterson S. E. Cutting loose. Abscission and dehiscence in Arabidopsis. Plant Physiol. 126, 494–500 (2001). 11402180 10.1104/pp.126.2.494 PMC1540116

[b19] Roberts J. A., Elliott K. A. & Gonzalez-Carranza Z. H. Abscission, dehiscence, and other cell separation processes. Annu. Rev. Plant Biol. 53, 131–158 (2002). 12221970 10.1146/annurev.arplant.53.092701.180236

[b20] Meir S., Hunter D. A., Chen J. C., Halaly V. & Reid M. S. Molecular changes occurring during acquisition of abscission competence following auxin depletion in Mirabilis jalapa. Plant Physiol. 141, 1604–1616 (2006). 16778017 10.1104/pp.106.079277 PMC1533941

[b21] Shi C. L. *et al.* Arabidopsis class I KNOTTED-like homeobox proteins act downstream in the IDA-HAE/HSL2 floral abscission signaling pathway. Plant Cell 23, 2553–2567 (2011). 21742991 10.1105/tpc.111.084608 PMC3226213

[b22] Estornell L. H. *et al.* The IDA Peptide Controls Abscission in Arabidopsis and Citrus. Front. Plant Sci. 6, (2015). 10.3389/fpls.2015.01003 PMC4652038 26635830

[b23] Sto I. M. *et al.* Conservation of the abscission signaling peptide IDA during Angiosperm evolution: withstanding genome duplications and gain and loss of the receptors HAE/HSL2. Front. Plant Sci. 6, 1152–1158 (2015). 26579174 10.3389/fpls.2015.00931 PMC4627355

[b24] Mitra S. K., Pereira L. S., Pathak P. K. & Majumdar D. Fruit abscission pattern of lychee cultivars. Acta Hortic. 665, 215–218 (2005).

[b25] Roe D. J., Menzel C. M., Oosthuizen J. H. & Doogan V. J. Effects of Current CO2 Assimilation and Stored Reserves On Lychee Fruit Growth. J. Hortic. Sci. 72, 397–405 (1997).

[b26] Stern R. A., Kigel J., Tomer E. & Gazit S. Mauritius lychee fruit-development and reduced abscission after treatment with the auxin 2,4,5-TP. J. Am. Soc. Hortic. 120, 65 (1995).

[b27] Yuan R. & Huang H. Litchi fruit abscission: its patterns, effect of shading and relation to endogenous abscisic acid. Sci. Hortic. 36, 281–292 (1988).

[b28] Xiang X., Qiu Y. & Zhang Z. Endogenous Hormones in the Fruit of Litichi chinensis cv. Nuomici Relating to Furit Abscission. J. Fruit Sci (1995).

[b29] Jian-Guo L. I. & Liu S. Z. Changes in Endogenous Polyamine Contents During Fruit Development of Litchi (Litchi chinensis). Plant Physiol. Commun. 40, 153–156 (2004).

[b30] Yuan R. Improvement of fruit-set in litchi chinensis sonn. through regulation of source-sink relationships. J. South China Agric. Univ (1992).

[b31] Yuan R. & Huang H. Regulation of Root and Shoot Growth and Fruit-dorp of Young Litchi Trees by Trunk Girdling in View of Source-Sink Relationships. J. Fruit Sci (1993).

[b32] Zhou X., Huang D., Huang H. & Dingyao A. W. Carbohydrate and Endohormone Status in Relation to Fruit Set as Influenced by Trunk Spiral Girdling of Young Litchi Trees. Acta Hortic. Sinica (1999).

[b33] Hieke S., Menzel C. M., Doogan V. J. & Lüdders P. The relationship between yield and assimilate supply in lychee (Litchi chinensis Sonn.). J. Hortic. Sci. Biotech. 77, 326–332 (2002).

[b34] Chang J. C. & Lin T. S. Gas exchange in litchi under controlled and field conditions. Sci. Hortic. 114, 268–274 (2007).

[b35] Yuan W. Q. *et al.* Seasonal Changes in Carbon Nutrition Reserve in Nuomici Litchi Trees and Its Relation to Fruit Load. Acta Hortic. Sinica (2009).

[b36] Li C. *et al.* An improved fruit transcriptome and the identification of the candidate genes involved in fruit abscission induced by carbohydrate stress in litchi. Front. Plant Sci. 6, 439 (2015). 26124768 10.3389/fpls.2015.00439 PMC4466451

[b37] Li C., Wang Y., Ying P., Ma W. & Li J. Genome-wide digital transcript analysis of putative fruitlet abscission related genes regulated by ethephon in litchi. Front. Plant Sci. 6, 1937–1942 (2015). 10.3389/fpls.2015.00502 PMC4493771 26217356

[b38] Agusti J., Merelo P., Cercos M., Tadeo F. R. & Talon M. Comparative transcriptional survey between laser-microdissected cells from laminar abscission zone and petiolar cortical tissue during ethylene-promoted abscission in citrus leaves. BMC Plant Biol. 9, 1–20 (2009). 19852773 10.1186/1471-2229-9-127 PMC2770498

[b39] Zhang J. Z., Zhao K., Ai X. Y. & Hu C. G. Involvements of PCD and changes in gene expression profile during self-pruning of spring shoots in sweet orange (Citrus sinensis). BMC Genomics 15, 1–16 (2014). 25308090 10.1186/1471-2164-15-892 PMC4209071

[b40] Botton A. *et al.* Signaling pathways mediating the induction of apple fruitlet abscission. Plant Physiol. 155, 185–208 (2011). 21037112 10.1104/pp.110.165779 PMC3075760

[b41] Zhu H. *et al.* Transcriptomics of shading-induced and NAA-induced abscission in apple (Malus domestica) reveals a shared pathway involving reduced photosynthesis, alterations in carbohydrate transport and signaling and hormone crosstalk. BMC Plant Biol. 11, 1–20 (2011). 22003957 10.1186/1471-2229-11-138 PMC3217944

[b42] Gilamado J. A. & Gomezjimenez M. C. Transcriptome analysis of mature fruit abscission control in olive. Plant & Cell Physiol. 54, 244–269 (2013). 23292600 10.1093/pcp/pcs179

[b43] Tadeo F. R. *et al.* “To fall or not to fall, that’s the question!” molecular mechanisms underlying organ abscission in citrus. Acta Hortic. 1189–1195 (2015).

[b44] Clough S. J. & Bent A. F. Floral dip: a simplified method for Agrobacterium-mediated transformation of Arabidopsis thaliana. Plant J. 16, 735–743 (1998). 10069079 10.1046/j.1365-313x.1998.00343.x

[b45] Sundaresan S. *et al.* Abscission of flowers and floral organs is closely associated with alkalization of the cytosol in abscission zone cells. J. Exp. Bot. 66, 913–919 (2014). 10.1093/jxb/eru483 PMC4339595 25504336

[b46] Schroder F., Lisso J., Lange P. & Mussig C. The extracellular EXO protein mediates cell expansion in Arabidopsis leaves. BMC Plant Biol. 9, 1–12 (2009). 19216774 10.1186/1471-2229-9-20 PMC2661892

[b47] Delk N. A., Johnson K. A., Chowdhury N. I. & Braam J. CML24, regulated in expression by diverse stimuli, encodes a potential Ca2+ sensor that functions in responses to abscisic acid, daylength, and ion stress. Plant Physiol. 139, 240–253 (2005). 16113225 10.1104/pp.105.062612 PMC1203374

[b48] Brown K. M. Ethylene and abscission. Physiol. Plantarum 100, 567–576 (1997).

[b49] Kuang J. F. *et al.* Carbohydrate stress affecting fruitlet abscission and expression of genes related to auxin signal transduction pathway in litchi. Int. J. Mol. Sci. 13, 16084–16103 (2012). 23443112 10.3390/ijms131216084 PMC3546680

[b50] Yoo S. D., Cho Y. H. & Sheen J. Arabidopsis mesophyll protoplasts: a versatile cell system for transient gene expression analysis. Nat. Protoc. 2, 1565–1572 (2007). 17585298 10.1038/nprot.2007.199

[b51] Zhong H. Y. *et al.* Selection of reliable reference genes for expression studies by reverse transcription quantitative real-time PCR in litchi under different experimental conditions. Plant Cell Rep. 30, 641–653 (2011). 21301853 10.1007/s00299-010-0992-8

[b52] Livak K. J. & Schmittgen T. D. Analysis of relative gene expression data using real-time quantitative PCR and the 2(-Delta Delta C(T)) Method. Methods 25, 402–408 (2001). 11846609 10.1006/meth.2001.1262

