# Supplementary material for: Identification and molecular characterization of an IDA-like gene from litchi, LcIDL1, whose ectopic expression promotes floral organ abscission in Arabidopsis
Source: Sci Rep. 2016 Nov 15;6:37135. doi: 10.1038/srep37135 (PMC5109030; doi:10.1038/srep37135)
Supplement: Supplementary Information [file srep37135-s1.doc]

**Identification and molecular characterization of an IDA-like gene from litchi, *LcIDL1*, whose ectopic expression promotes floral organ abscission in *Arabidopsis***

Peiyuan Ying^1,2^, Caiqin Li^1,2^, Xuncheng Liu^3^, Rui Xia^1,2^, Minglei Zhao ^1,2^,^*^ and Jianguo Li^1,2,*^

1 State Key Laboratory for Conservation and Utilization of Subtropical Agro-Bioresources, China Litchi Research Center, South China Agricultural University, Guangzhou, 510642, China.

2 Guangdong Litchi Engineering Research Center, College of Horticulture, South China Agricultural University, Guangzhou, 510642, China.

3 Key Laboratory of Plant Resources Conservation and Sustainable Utilization, South China Botanical Garden, Chinese Academy of Sciences, Guangzhou 510650, China

* Corresponding author, E-mail:zhaominglei503@126.com; jianli@scau.edu.cn

**Supplemental Table. Primers used in this study**

**Primer name (5′-3′) Purpose**

LcIDL1-For1: ATGGCTTCTAAAGCTATGCA For qRT-PCR in litchi

LcIDL1-Rev1: CCAGTGAGTCTTCCTGGTCT For qRT-PCR in litchi

LcIDL1-For2: GTTCGAGACGGGTTTCCAATA For qRT-PCR in Arabidopsis

LcIDL1-Rev2: TGTGCCCATTAACATCACCAT For qRT-PCR in Arabidopsis

LcIDL1-35S-For: GGACTCTTGACCATGGTAATGGCTTCTAAAGCTATGCATC For 35S:LcIDL15 construction

LcIDL1-35S- Rev: GTCAGATCTACCATGGTAATCAGACGGTGTAGAATCCA For 35S:LcIDL15 construction

LcIDL1-native-For: ATCCTCTAGAGTCGACATGGCTTCTAAAGCTATGCATC For pAtIDA:LcIDL15 construction

LcIDL1-native- Rev: GTCAGATCTACCATGGTAATCAGACGGTGTAGAATCCA For pAtIDA:LcIDL15 construction

AtIDA -35S-For: GGACTCTTGACCATGGTAATGGCTCCGTGTCGTACGATG For 35S: AtIDA construction

AtIDA -35S-Rev: GTCAGATCTACCATGGTATGAGGAAGAGAGTTAACAAAAGAGTTG For 35S: AtIDA construction

AtIDA-promoter-For: ATCCTCTAGAGTCGACAACCCTCGTTCTGAATCAAAGGGT For pAtIDA:LcIDL15 construction

AtIDA-promoter-Rev: TAGAAGCCATGTCGACTTGGTAGTCAATGTTTTTTTTCTTC For pAtIDA:LcIDL15 construction

ida-2-LP: TTTTGGCCACTTGAGAAATTG For genotyping of ida-2

ida-2-RP: GAAAATAAAAGTCGAAGGCGG For genotyping of ida-2

LBb1.3: ATTTTGCCGATTTCGGAAC For genotyping of ida-2

AtUBQ-For: GATCTTTGCCGGAAAACAATTGGAGGATGGT For qRT-PCR in Arabidopsis

AtUBQ-Rev: CGACTTGTCATTAGAAAGAAAGAGATAACAGG For qRT-PCR in Arabidopsis

GAD4-For: CGTCGGAGGCGATTATGTTG For qRT-PCR in Arabidopsis

GAD4-Rev: CTTGCGAATTTCTCCCAGCA For qRT-PCR in Arabidopsis

TCH4-For: TGCTTCTTACCGTGGCTTTC For qRT-PCR in Arabidopsis

TCH4-Rev: TCTGCACCCATCTCATCCTT For qRT-PCR in Arabidopsis

EXO-For: AAACTCGCAACACCCAGCAA For qRT-PCR in Arabidopsis

EXO-Rev: CCCGCAACGACTCATACCAA For qRT-PCR in Arabidopsis

EXL1-For: TATTGTTCGCTGTTGCTTTC For qRT-PCR in Arabidopsis

EXL1-Rev: TACTTCTCCACCGTCTTCCA For qRT-PCR in Arabidopsis

EXL1-For: TATTGTTCGCTGTTGCTTTC For qRT-PCR in Arabidopsis

EXL1-Rev: TACTTCTCCACCGTCTTCCA For qRT-PCR in Arabidopsis
